# Perceptions of Heated Tobacco Products (HTPs) and Intention to Quit Among Adult Tobacco Users in Korea

**DOI:** 10.2188/jea.JE20200213

**Published:** 2022-08-05

**Authors:** Jinju Park, Han Joo Kim, Sang Hwa Shin, Eunjung Park, Jin-Kyoung Oh, Eun Young Park, Min Kyung Lim

**Affiliations:** 1 Department of Cancer Control and Population Health, Graduate School of Cancer Science and Policy, National Cancer Center, Goyang, Republic of Korea; 2 Division of Cancer Prevention & Early Detection, National Cancer Control Institute, National Cancer Center, Goyang, Republic of Korea

**Keywords:** heated tobacco products, IQOS, harm reduction, intention to quit, advertising and promotion

## Abstract

**Background:**

The popularity of heated tobacco products (HTPs) has been growing globally but, limited information exists on tobacco use behaviors and its impact on tobacco control. This study investigates awareness and perception of HTPs among tobacco users and whether perceptions of HTPs are associated with HTP use and intention to quit.

**Methods:**

We invited 2,000 tobacco users aged 19–65 years with countrywide representation to an online survey in November 2018. Information on general characteristics, tobacco use behaviors, awareness and perception of HTPs, and intention to quit were gathered. Multinomial logistic regression analysis and ANCOVA were used for estimation of association and comparison.

**Results:**

Among all tobacco users, 36.8% were classified as ever users, whereas 28.3% had used HTPs in the past 30 days, which was higher than expected. Users of liquid-based e-cigarettes (odds ratio [OR] 1.578; 95% confidence interval [CI], 1.210–2.056) and poly-product users (OR 2.029; 95% CI, 1.121–3.671) showed higher intention to quit within 1 month when compared to users of conventional cigarettes (CCs), whereas HTP users and dual product users did not. HTP users rated HTPs more favorably than CCs in terms of smoke, smell, harm, aid in quitting, design, and price than users of other products did (*P*-value < 0.001).

**Conclusion:**

We find that positive perception of HTPs following strategic marketing from tobacco companies could have contributed to a greater increase in HTP use than expected in Korea. However, HTPs might not be considered substitutes for CCs for quitting tobacco use because a significant proportion of dual product users reported a lower intention to quit.

## INTRODUCTION

The introduction and growing use of the novel tobacco products (NTPs) have been an emerging problem in tobacco control due to the renormalization of tobacco use, with companies marketing NTPs as products to reduce harm or to help quit. This has resulted in a novel challenge for tobacco control.^1^^,^^2^ Furthermore, NTPs have been rapidly introduced and aggressively promoted in countries or regions where tobacco control policies, such as taxation and smoke-free policies, have been implemented or enforced. Therefore, knowledge dissemination with continual gathering of evidence on NTPs is urgently required to deploy tobacco regulation, appropriately.

In Korea, conventional cigarettes (CCs) are still the predominant tobacco product. Following the enforcement of smoke-free policies in 2013 and tax increases in 2015, liquid-based e-cigarettes (LECs), snus, and heated tobacco products (HTPs) were rapidly introduced in 2011, 2013, and 2017, respectively. Snus has not been very popular in Korea, and LECs use had increased slightly upon introduction but has decreased since 2015 (prevalence in 2018: Snus 0.2%, LEC 3.6%).^3^ However, HTP use has spread dramatically in Korea since its introduction in 2017.^3^ As estimated by Euromonitor, about 14 out of 100 (13.6%) smokers in 2019 and 28 out of 100 (27.8%) in the next 5 years will use HTPs compared to just 3 out of 100 (3%) in 2017.^4^ Increased use of HTPs in Korea may be linked to the marketing of HTPs by tobacco companies as a safer alternative to CCs, with Philip Morris International announcing that the goal of the IQOS tobacco heating system is “to switch smokers to less harmful alternatives”. Additionally, HTPs have been promoted as high-tech smokeless devices, providing the benefit of smoking with less odor and ash.^5^^–^^7^

Aggressive marketing, coupled with the growing use of HTPs, could renormalize tobacco use while nullifying the current control efforts in Korea. Moreover, their global popularity is growing, with marketing strategies specifically tailored to the social and cultural factors at play in each country or region. However, necessary information to examine consumer awareness, perception, and attitudes toward HTPs and how these impact worldwide control efforts to aid people recovering from tobacco addiction is currently lacking.

The current study was designed to investigate awareness and perception of HTPs among tobacco users and whether those perceptions are associated with HTP use and intention to quit. This study aims to fill the gap in our understanding of the empirical basis for transitioning tobacco product use at the population level, thus determining the best approaches to attain the ultimate goal of tobacco control.

## METHODS

### Study procedures

In November 2018, we carried out an online survey to identify the prevalence of HTP use, awareness, and perception among adult tobacco users in Korea. We recruited 2,000 any type of tobacco product users (264 female and 1,736 male) aged 19–65 years who were enrolled in the Tillion Internet research panel managed by the survey company Panel Marketing Interactive (www.pmirnc.com). The Tillion panel is the largest research panel in Korea and comprises a diverse group of over 30 million OK cashback members in Korea, which is a payback membership including most members of Korean society. Thus, the Tillion panel is an inclusive survey target group that represents the Korean population across different regions and economic classes. Participants were recruited to the study via an email invitation. To avoid possible selection bias, proportionate stratified sampling was used to approximate the gender, age, and residential area distributions using data from the Korea National Health and Nutrition Examination Survey (KNHANES) (2017).^8^

This study has been carried out in accordance with Declaration of Helsinki. The study protocol was approved by the Institutional Review Boards of the National Cancer Center (NCC2018-0269). During the survey, online informed consents were obtained from all participants.

### Measures

General characteristics of the participants, including gender (male or female), age (19–29, 30–39, 40–49, 50–59, or 60–64 years), residential area (urban or rural), occupation (technical/professional/office, service and sales/production management, or others), and marital status (single or married) were included.

Smoking behaviors examined in the study included frequency of CCs smoking (cccasiona or daily), and numbers of CCs smoked per day (<10 or ≥10). To measure awareness and use of HTPs, two questions were asked as the order listed: “Have you ever used HTPs?” (yes or no) and “Which of the following products have you ever used as HTPs?” (LECs and HTPs eg, IQOS, glo, lil). For the second question, each brand name of HTPs and LECs was suggested as an option together with its image to ensure that study participants chose the exact tobacco products they have used and to correct any potential confusion. To determine whether there was any confusion between LECs and HTPs, the results were compared with the answer from the question “Have you ever used HTPs?” (yes or no).

After the correction of any potential confusion between LECs and HTPs, participants were asked to answer the following questions to determine the prevalence of tobacco use by the type of tobacco product: “Which of the following products did you use in the past 30 days? (CCs, LECs, HTPs, CCs & LECs, CCs & HTPs, LECs & HTPs, CCs & LECs & HTPs)”.

Six different statements were employed to assess awareness and perceptions of HTPs: Compared to CCs, (1) I think HTPs have no smell; (2) I think HTPs have no smoke; (3) I think HTPs have no secondhand smoke (SHS) exposure; (4) I think HTPs are helpful to quit smoking; (5) I think HTPs are attractive and appealing (including device); and (6) I think the price of HTPs is affordable. The response scale score ranged from 1 to 7, with lower scores indicating positive perceptions of HTPs.

Intention to quit-related questions were asked using the following phrasing: “Do you have a plan to quit use of tobacco products?” (Do not have a plan to quit within 6 months/Do have a plan to quit within 6 months/Do have a plan to quit within 1 month). Intention to quit applies to all type of tobacco products including the products in use.

### Statistical analysis

Chi-square tests were performed to examine associations between types of tobacco use and the intention to quit within 1 month or 6 months. A multivariable logistic regression analysis was performed to evaluate the factors related to the intention to quit within 1 month. Furthermore, frequency weighted statistical analysis by the variable of occupation was applied for minimizing sampling bias between our data and KNHANES data.^8^ ANCOVA and *F*-test were used to compare mean scale scores of perceptions of HTPs grouped according to types of tobacco use. All analyses were performed using SPSS software, version 21.0 (IBM, Armonk, NY, USA) and a *P*-value of <0.05 was considered to indicate statistical significance.

## RESULTS

To the question “Have you ever used HTPs?”, 33.3% of participants who answered “yes” were re-classified as ever LEC users, because they chose an image of an LEC as the HTP used. After this correction, 36.8% of total tobacco users were classified as ever HTP users.

Table 1 depicts the types of tobacco product use in the past 30 days and the intention to quit within 1 month or 6 months among the participants. Most participants were CC users (61.3%), followed by those who were dual- or poly-product users (18.6%), HTP users (13.8%), and LEC users (6.3%). Of the users with an intention to quit, 1,040 (52.0%) and 380 (19.0%) of 2,000 smokers intended to quit within 6 months and 1 month, respectively. The proportion of users with an intention to quit within 1 month was highest among LEC users (30.9%) and lowest among HTP users (15.9%).

**Table 1.** Intention to quit by the type of tobacco product used among adult tobacco users in Korea

Types of tobacco used in the past 30 days	Total (*n* = 2,000)	Intention to quit within 6 months (*n* = 1,040)	Intention to quit within 1 month (*n* = 380)
CC	1,257 (61.3%)	611 (48.8%)	240 (19.3%)
LEC	128 (6.3%)	69 (55.2%)	40 (30.9%)
HTPs	259 (13.8%)	147 (58.0%)	41 (15.9%)
Dual & Poly	356 (18.6%)	213 (59.6%)	59 (16.7%)
CC+LEC	84 (4.2%)	58 (68.6%)	8 (11.0%)
CC+HTPs	228 (12.2%)	133 (57.6%)	39 (16.9%)
LEC+HTPs	23 (1.2%)	12 (58.6%)	4 (17.2%)
CC+LEC+HTPs	21 (1.1%)	10 (49.1%)	8 (35.8%)

CC, conventional cigarette; HTPs, heated tobacco products; LEC, liquid-based e-cigarette.

After adjustment for all the appropriate variables shown in Table 2, LEC users (odds ratio [OR] 1.578; 95% confidence interval [CI], 1.210–2.056) and poly-product users (OR 2.029; 95% CI, 1.121–3.671) were found to have a significantly higher intention to quit within 1 month compared to CC users. While HTP users (OR 0.797; 95% CI, 0.634–1.003) showed a lower intention to quit within 1 month, although it was not significantly different from CC users, dual product users (OR 0.766; 95% CI, 0.621–0.946) showed a significantly lower intention to quit within 1 month. Smokers aged 60 or above and daily smokers (OR 0.388; 95% CI, 0.309–0.488) had the least intention to quit within 1 month (Table 2).

**Table 2.** Type of tobacco product used and other factors associated with intention to quit within 1 month

		Intention to quit within 1 month					

		Total *n* = 2,000	*n* (%)	Crude		Multi-adjusted^a^	

				OR	95% CI	OR	95% CI
Gender	Female	264 (13.2)	58 (22.0)	Ref	Ref	Ref	Ref
	Male	1,736 (86.8)	322 (18.5)	0.802	(0.655∼0.981)	1.142	(0.919∼1.419)

Age, years	19–29	430 (21.5)	107 (24.9)	Ref	Ref	Ref	Ref
	30–39	534 (26.7)	102 (19.1)	0.707	(0.580∼0.862)	0.820	(0.655∼1.028)
	40–49	491 (24.6)	89 (18.1)	0.678	(0.553∼0.831)	0.866	(0.677∼1.108)
	50–59	443 (22.2)	67 (15.1)	0.551	(0.443∼0.686)	0.670	(0.509∼0.881)
	60–64	102 (5.1)	15 (14.7)	0.612	(0.422∼0.886)	0.618	(0.408∼0.934)

Area of residence	Rural	1,093 (54.7)	200 (18.3)	Ref	Ref	Ref	Ref
	Urban	907 (45.4)	180 (19.8)	1.115	(0.969∼1.284)	1.115	(0.969∼1.284)

Occupation	Technical/Professional/Office	1,297 (64.9)	252 (19.4)	Ref	Ref	Ref	Ref
	Service and Sales/Production management	445 (22.3)	76 (17.1)	0.854	(0.705∼1.035)	0.859	(0.704∼1.049)
	Others^b^	258 (12.9)	52 (20.2)	1.047	(0.764∼1.433)	0.818	(0.585∼1.143)

Current marital status	Single	847 (42.4)	179 (21.1)	Ref	Ref	Ref	Ref
	Married	1,153 (57.7)	201 (17.4)	0.790	(0.685∼0.911)	0.915	(0.762∼1.099)

Frequency of CC smoking	Occasional	542 (27.1)	174 (32.1)	Ref	Ref	Ref	Ref
	Daily	1,458 (72.8)	206 (14.1)	0.350	(0.302∼0.406)	0.388	(0.309∼0.488)

Numbers of CC smoked per day	<10	1,165 (58.3)	218 (26.1)	Ref	Ref	Ref	Ref
	≥10	835 (41.8)	162 (13.9)	0.452	(0.392∼0.521)	0.900	(0.717∼1.129)

Types of tobacco used in the past 30 days	CC	1,257 (62.9)	240 (19.1)	Ref	Ref	Ref	Ref
	LEC	128 (6.4)	40 (31.3)	1.876	(1.455∼2.420)	1.578	(1.210∼2.056)
	HTPs	259 (13.0)	41 (15.8)	0.796	(0.637∼0.993)	0.797	(0.634∼1.003)
	Dual^c^	335 (16.8)	51 (15.2)	0.770	(0.629∼0.943)	0.766	(0.621∼0.946)
	Poly^d^	21 (1.1)	8 (38.1)	2.343	(1.327∼4.137)	2.029	(1.121∼3.671)

CC, conventional cigarette; CI, confidence interval; HTPs, heated tobacco products; LEC, liquid-based e-cigarette; OR, odds ratio.

^a^Adjusted for gender, age, area of residence, occupation, current marital status, frequency of CC smoking, numbers of CC smoked per day, types of tobacco used in the past 30 days.

^b^Others (eg, student, housewife).

^c^Using two tobacco products (CC+LEC, CC+HTPs, LEC+HTPs).

^d^Using three tobacco products (CC+LEC+HTPs).

Participants were asked to score a set of statements assessing awareness and perceptions of HTPs compared to CCs shown in Table 3. HTP users rated HTPs more favorably across this set of measures when compared to CC, LEC, and dual- or poly-product users. The lowest mean score on the statements such as HTPs have no smell, no smoke, no SHS exposure, is helpful to quit, is attractive and appealing (including device), and the price of HTPs is affordable was identified in HTP users and was significantly different when compared to users of other types of tobacco products (*P*-value < 0.001).

**Table 3.** Perceptions of HTPs by the type of tobacco used

Items to measure awareness and perception of HTPs			Total mean (SD)	CC mean (SD)	LEC mean (SD)	HTPs mean (SD)	Dual/Poly^a^ mean (SD)	*P*-value^b^ (*F*-value, df)	*P*-value^c^
**have no smell (1)**	⟶	**have smell (7)**	4.37 (2.215)	4.58^*^ (2.146)	4.94^*^ (2.24)	3.62^†^ (2.281)	3.95^†^ (2.214)	<0.001 (*F* = 21.231, df = 3,1996)	<0.001
**have no smoke (1)**	⟶	**have a smoke (7)**	5.17 (2.115)	5.3^*^ (2.098)	5.92^‡^ (1.971)	4.68^†^ (2.127)	4.81^†^ (2.092)	<0.001 (*F* = 15.436, df = 3,1996)	<0.001
**no SHS exposure (1)**	⟶	**there is SHS exposure (7)**	5.25 (2.182)	5.5^*^ (2.152)	5.52^*^ (2.066)	4.52^†^ (2.207)	4.79^†^ (2.127)	<0.001 (*F* = 21.610, df = 3,1996)	<0.001
**helpful to quit (1)**	⟶	**not helpful to quit (7)**	5.94 (2.240)	6.18^*^ (2.218)	5.56^†^ (1.894)	5.39^†^ (2.301)	5.64^†^ (2.270)	<0.001 (*F* = 13.594, df = 3,1996)	<0.001
**product is attractive (1)**	⟶	**product is not attractive (7)**	5.51 (2.031)	5.69^*^ (1.984)	5.58^*^ (2.068)	4.78^†^ (2.084)	5.37^*^ (2.022)	<0.001 (*F* = 15.339, df = 3,1996)	<0.001
**price is affordable (1)**	⟶	**price is not affordable (7)**	6.36 (2.084)	6.51^*^ (2.056)	6.13^*^ (1.932)	5.89^†^ (2.266)	6.28^†^ (2.043)	<0.001 (*F* = 7.211, df = 3,1996)	0.001

CC, conventional cigarette; HTPs, heated tobacco products; LEC, liquid-based e-cigarette; S.D, standard deviation; SHS, secondhand smoke.

Note: The response scale score ranged from 1 to 7, with lower scores indicating a favorable perceptions and positive attitudes toward HTP.

^a^Using more than two tobacco products (CC+LEC, CC+HTPs, LEC+HTPs, CC+LEC+HTPs).

^b^*P*-values were calculated by One-way ANOVA.

^c^*P*-values were calculated by ANCOVA adjusted for gender, age, area of residence, occupation, current marital status, frequency of CC smoking, numbers of CC smoked per day.

^*,†,‡^Identification of same group was determined by Duncan’s Multiple Range Test (same group presented by same symbol).

## DISCUSSION

Our study sought to determine the extent of HTP awareness and perceptions of adult tobacco users in Korea after the recent introduction and promotion of HTPs. Furthermore, we also investigated how HTP use can impact the intention to quit, which in turn may be a predictor of HTP use and the trajectory of tobacco prevalence in the future.

When probing the type of tobacco used in the past 30 days, our study found that 13.8% of all tobacco users were users of HTPs only (Table 1). When combined with the 14.5% dual- or poly-product users that use HTPs as one of the products, the proportion of HTP users comprises 28.3% of adult tobacco users in this survey. This is a substantial increase from an earlier Euromonitor data recorded when HTPs were first introduced in Korea (3% of tobacco users, 2017). It is also higher than the numbers reported by the KNHANES in 2018 (17.5% of tobacco users were classified as HTP users) and estimates from the recent Euromonitor report in 2019 (13.6% of tobacco users).^3^^,^^4^ The results of our study indicate that HTP use has rapidly increased and is more prevalent than we expected in Korea.

**Figure 1.**
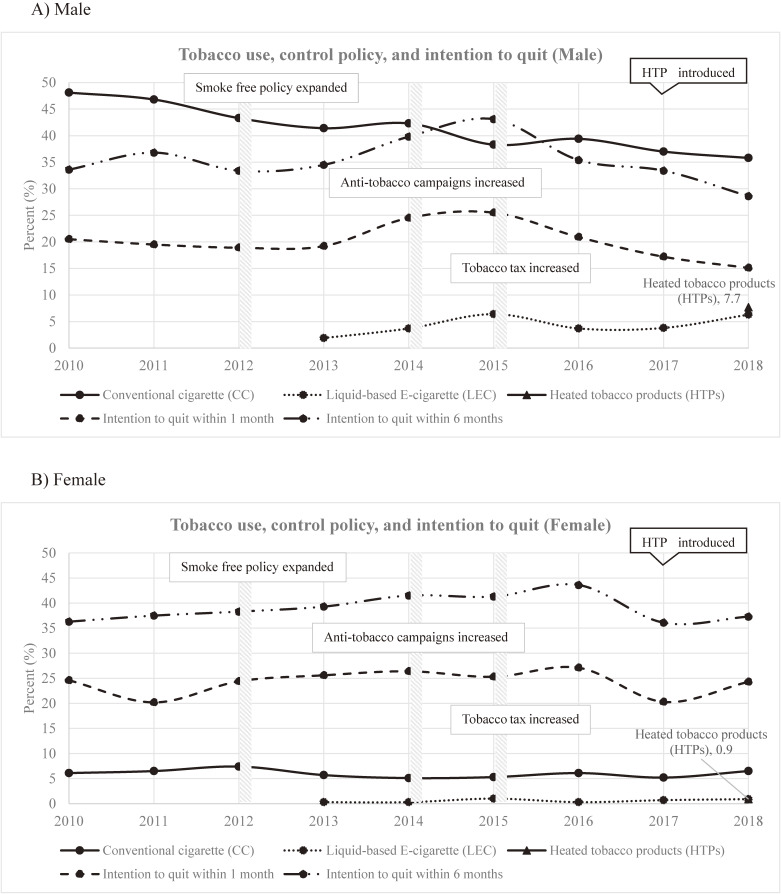
Prevalence of tobacco use and intention to quit under the change of tobacco control policies in Korea for (A) males and (B) females. Data source: Korea Centres for Disease Control and Prevention. Korean National Health and Nutrition Examination Survey. 2018. http://knhanes.cdc.go.kr/knhanes/main.do (accessed 29 Jan 2020).

This increase in HTP use might be explained by aggressive promotion and various marketing tactics employed by tobacco companies. As reported by a previous study in Korea, tobacco companies used one of the largest convenience store chains to sell IQOS.^9^ Companies also promoted HTPs on the internet by releasing YouTube videos to demonstrate the use of HTPs and by offering discount coupons to customers who registered on the promotional website.^10^^,^^11^ Flagship stores similar to famous electronic stores were opened and marketed HTPs as ‘a high-tech product different from regular cigarettes’.^6^ Thus, proliferation of HTP-related advertising and promotion via existing and new channels could have increased the desirability of HTPs to users. Tobacco companies also improved perception of HTPs by marketing them as 1) less harmful products than CCs, 2) smokeless and odorless alternatives to CCs, 3) aids to quit and replace CCs, and 4) a high-tech device to be favored.^2^^,^^12^^,^^13^ These marketing strategies may have increased the interest in HTPs and improved the perception of the products, thus resulting in the increased consumption of HTPs. Our study found more positive perceptions of HTPs, particularly among HTP users, who rated HTPs more favorably when compared to CCs. HTP users perceived HTPs as less likely to have odor, smoke, and SHS exposure compared to CCs. They were more likely to think HTP use is helpful to quit tobacco use, attractive and appealing in design of the products and devices, and affordable in price (Table 3). In the context of current social control practices, where smoke-free areas have been expanded and enforced, promotion of HTPs as products without smoke and odor could encourage smokers and non-smokers to use HTPs and even encourage the perception that HTPs are permissive products in smoke-free areas. Similar results and explanations were suggested by a previous study that investigated the effect of positive perception on the consumption of HTPs.^14^ This positive perception led by aggressive marketing might be reinforced by the greater accessibility and affordability of the products. Furthermore, the stringent political and legal control policies on CC smoking in Korea have not been extended to NTPs, which include HTPs. Importantly, because HTPs do not meet the legal definition of a tobacco product in Korea, their promotion and sales have evaded public health restrictions imposed by tobacco control laws.^15^ In addition, HTPs were named and marketed as a “conventional cigarette like e-cigarettes” when they were introduced, leading people to confuse them with LECs that have been considered less harmful and advertised as cessation aids in Korea since 2007.^16^^,^^17^ Findings from our study, using images to confirm the tobacco product used, confirmed that some HTP users were actually LEC users. Similarly, an increase in LEC use has also been reported since 2017 in the KNHANES, even though there had been a downward trend before (Figure 1).^3^ Therefore, it is likely that the increased consumption of HTPs partially results from the ambiguity of the product name and perceptions associated with it.

The other main finding of our study is that HTPs should neither be considered cessation aids nor substitutes for CCs. As shown in Table 2, HTP use and dual-product use lowered the intention to quit within 1 month, even though this decrease was not statistically significant. HTP users without intention to quit within 1 month had a more favorable perception of HTPs in each category and these results were similar in other tobacco product users (eTable 1). As reported by KNHANES, the intention to quit within 1 month increased among male tobacco users upon strengthening of the tobacco control policy in 2014, but it has been turned down in 2016 and then lowered in 2017 than in past years before strengthening of tobacco control policies (Figure 1). Contrary to the tobacco industry’s claim that smokers can switch from CCs to HTPs and then go on to quit tobacco use, we found that around 28% of tobacco users were HTP users, and 14.5% of tobacco users used HTPs in addition to other tobacco products. Similarly, a study investigating IQOS use among adolescents found that most were poly-tobacco product users of CCs and/or LECs in Korea.^18^ Another Korean study reported that none of the current users of IQOS had switched from CCs to IQOS.^9^ Thus, it appears that the availability of HTPs can impair the motivation to quit tobacco product use or encourage smokers to start using NTPs.

In Korea, stringent tobacco control measures, such as expanded smoke-free areas, tax increases, and labeling on packages, have been implemented together with sweeping mass media campaigns on the detrimental impact of tobacco use on health (Figure 1).^19^ The enforcement of tobacco control policies has resulted in a 4% decrease of CC smoking but may have encouraged tobacco companies to introduce alternative products, such as NTPs, including HTPs, in Korea. After the introduction of manufactured cigarettes in Korea, the most prevalent tobacco product was CCs, while others were rarely used.^7^ However, snus, LECs, and HTPs have been introduced upon the enforcement of tobacco control policies. HTP use has rapidly increased and become more prevalent following aggressive marketing and is preferred by CC smokers, whereas snus failed to gain popularity, and LEC use spiked at first but has subsequently decreased since 2015. Given the increased consumption of NTPs, strong curbs on the marketing and sale of NTPs, including HTPs, should be enforced with comprehensive legal regulations and monitoring of NTP use in Korea. These measures will likely impact the population’s attitude toward HTPs and user behavior.^20^^–^^22^ Stringent control strategies should also be considered in other countries where NTPs are due to be launched.

Although our study was one of the first to investigate the perception and prevalence of HTPs and suggest a potential impact of HTP use on the intention to quit among Koreans, there are several limitations. First, information on socioeconomic status, which might affect representativeness of study participants, perception on HTP, and type of tobacco products they used, could not be considered for sampling of participants and adjustment in multivariable analysis, although information on occupation was included. Second, with the cross-sectional design of the study, the association between HTP use and intention to quit could not be determined temporally. Third, the effect of single- or dual-product use of HTPs on tobacco cessation could not be measured. Fourth, poly-product use associated with higher intention to quit, which is different in dual-product use. As well, it could not be determined if dual- or poly-product HTP users represent individuals who are in the process of switching completely from CCs to HTPs or on the path to complete cessation. Longitudinal studies with larger sample are required in the future to evaluate these causal inferences. Although our study identified the type of tobacco used by the consumer during the last 30 days, detailed information on tobacco use behaviors, such as frequency and amount, was not gathered. Therefore, occasional users of each type of tobacco product were classified in the same category as continuous users. This could affect the association between the type of tobacco used and the intention to quit.

## SUPPLEMENTARY MATERIAL

eTable 1. Perception toward HTPs and the intention to quit by the type of tobacco product used
