# Supplementary material for: Perceptions of Heated Tobacco Products (HTPs) and Intention to Quit Among Adult Tobacco Users in Korea
Source: J Epidemiol. 2022 Aug 5;32(8):357–62. doi: 10.2188/jea.JE20200213 (PMC9263615; doi:10.2188/jea.JE20200213)
Supplement: Supplementary file 1 [file je-32-357-s001.pdf]

eTable 1. Perception toward HTPs and the intention to quit by the type of tobacco product used

Items to measure perception on HTPs	Total mean (SD)	CC		LEC		HTPs		Dual/Poly[a]	
		within 1 month	no or ≥1 month	within 1 month	no or ≥1 month	within 1 month	no or ≥1 month	within 1 month	no or ≥1 month
**have no smell (1) → have smell (7)**	4.37 (2.215)	4.73 (2.025)	5.03 (2.336)	4.75 (2.133)	4.54 (2.149)	4.17 (2.333)	3.52 (2.262)	3.95 (2.021)	3.95 (2.254)
**have no smoke (1) → have smoke (7)**	5.17 (2.115)	6.03 (2.006)	5.88 (1.964)	5.60 (2.067)	5.23 (2.10)	4.83 (2.19)	4.65 (2.118)	5.07 (2.108)	4.76 (2.089)
**no SHS exposure (1) → there is SHS exposure (7)**	5.25 (2.182)	5.98 (1.993)	5.32 (2.076)	5.76 (2.212)	5.43 (2.134)	4.73 (2.168)	4.48 (2.217)	5.02 (2.308)	4.75 (2.091)
**helpful to quit (1) → not helpful to quit (7)**	5.94 (2.240)	5.6 (1.919)	5.55 (1.893)	6.32 (2.285)	6.15 (2.201)	5.51 (2.099)	5.37 (2.34)	5.44 (2.493)	5.68 (2.226)
**product is attractive (1) → product is not attractive (7)**	5.51 (2.031)	5.55 (2.148)	5.59 (2.043)	5.73 (1.948)	5.68 (1.993)	4.85 (1.838)	4.77 (2.131)	5.41 (2.085)	5.36 (2.012)
**price is affordable (1) → price is not affordable (7)**	6.36 (2.084)	6.75 (1.864)	5.84 (1.905)	6.5 (1.985)	6.51 (2.074)	6.07 (1.992)	5.86 (2.317)	5.75 (2.098)	6.39 (2.019)

CC, conventional cigarette; HTPs, heated tobacco products; LEC, liquid-based e-cigarette; S.D, standard deviation; SHS, secondhand smoke.

[a] Using more than two tobacco products (CC+LEC, CC+HTPs, LEC+HTPs, CC+LEC+HTPs).
